# The Impact of Sport Training on Oral Health in Athletes

**DOI:** 10.3390/dj9050051

**Published:** 2021-05-03

**Authors:** Domenico Tripodi, Alessia Cosi, Domenico Fulco, Simonetta D’Ercole

**Affiliations:** Department of Medical, Oral and Biotechnological Sciences, University “G. D’Annunzio” of Chieti-Pescara, Via dei Vestini 31, 66100 Chieti, Italy; tripodi@unich.it (D.T.); alessiacosiac@gmail.com (A.C.); domenico.fulco90@gmail.com (D.F.)

**Keywords:** saliva, oral microbiota, S-IgA, mouthguards, oral disease, sports dentistry

## Abstract

Athletes’ oral health appears to be poor in numerous sport activities and different diseases can limit athletic skills, both during training and during competitions. Sport activities can be considered a risk factor, among athletes from different sports, for the onset of oral diseases, such as caries with an incidence between 15% and 70%, dental trauma 14–70%, dental erosion 36%, pericoronitis 5–39% and periodontal disease up to 15%. The numerous diseases are related to the variations that involve the ecological factors of the oral cavity such as salivary pH, flow rate, buffering capability, total bacterial count, cariogenic bacterial load and values of secretory Immunoglobulin A. The decrease in the production of S-IgA and the association with an important intraoral growth of pathogenic bacteria leads us to consider the training an “open window” for exposure to oral cavity diseases. Sports dentistry focuses attention on the prevention and treatment of oral pathologies and injuries. Oral health promotion strategies are needed in the sports environment. To prevent the onset of oral diseases, the sports dentist can recommend the use of a custom-made mouthguard, an oral device with a triple function that improves the health and performance of athletes. During training, the sports dentist must monitor the athletes and the sports examination protocol must be implemented with the inclusion of the clinical examination, quantitative and qualitative analysis of saliva and instructions on the use, cleansing and storage of the mouthguard.

## 1. Introduction

The growing diffusion of sports activities is focusing attention on the development of diseases correlated with the sporting performance, in addition to the health benefits.

The athlete should know the risks associated with carrying out sporting activities and direct more attention to the health status of his oral cavity since the alterations of the oral cavity contribute in a negative way to the health, well-being and physical performance of the athlete [1].

The main disorders of the oral cavity, correlated with the performance of various sports that have a significant impact on the quality of life of athletes, are represented by trauma, joint disorders, alterations and/or oral pathologies, such as caries, erosions, dental discolorations and periodontal disease. Moreover, the intense physical exercise at the start of competitions and throughout training time involves a reduction in salivary flow rate and in secretory IgA (S-IgA) load, resulting in a decrease in the host organism’s defenses and, therefore, an increase in susceptibility to particular pathologies, such as upper respiratory tract infections (URTI) and, specifically, pathologies of the oral cavity [2,3,4,5].

Therefore, during sport activities, the athletes must be monitored by sport dentists for changes affecting their oral cavity and the sports examination protocol must be implemented with the inclusion of clinical examination, quantitative and qualitative analyses of saliva and the instructions on the use, cleansing and storage of the mouthguard, which is a dental device used by athletes who need to wear oral protection during sports activities [6].

The first purpose of the present review is to describe the main diseases affecting the athletes’ oral cavity and the changes that occur in their oral ecosystem during sport training with and/or without use of mouthguard. The second objective is to delineate a program of prophylaxis and/or treatment for the diseases and oral changes reported.

## 2. The Oral Ecosystem and the Saliva Diagnostic Function

The oral ecosystem is a moist environment that includes the various anatomical microniches of the oral cavity and the oral microbiome. Two important physiological fluids, saliva and gingival crevicular fluid, wet the oral ecosystems and provide water, nutrients, adherence and antimicrobial factors [7].

Different physiochemical factors characterize the habitat of the oral cavity, thus supporting the development and growth of different microbial communities.

The oral microbiome includes a complex range of microorganisms, either present in saliva as organisms in the planktonic phase or, in the sessile phase, adhering to the oral surfaces as a dental plaque biofilm, namely 2000 taxa of bacteria, archaea and protozoa, fungi and viruses [7,8,9].

They are organized through a mechanism of mutual exchange of resources that give life to an ecosystem and, in most cases, they live in balance with each other and they bring significant benefits to the host organism. The major oral diseases, such as tooth decay and periodontal disease, arise from imbalance or dysbiosis within the plaque biofilm [7,8,9].

The gingival fluid is an exudate from plasma; it contains proteins, albumin, leukocytes, immunoglobulins and complement and is characterized by slow diffusion through the healthy gingiva which increases during inflammation [8].

Human saliva is a body fluid secreted by the salivary glands, whose function is to maintain the integrity of the hard and soft tissues of the oral cavity and to wet the mucous membranes of the mouth, throat and larynx in order to maintain the homeostasis of the oral ecosystem [10,11]. Normally, the daily salivary flow is estimated to be between 0.5 and 2 L [10]; the temperature of the saliva is more or less 35–36° and the pH is 7 ± 0.25, being saturated with calcium phosphates [11]. The buffer systems present in the saliva allow the maintenance of proper acid–base balance, swinging the pH values between 5.7 and 6.2 in the rest condition, to values of stimulated saliva of 8, depending on the habits of oral hygiene, food and the buffering action of saliva [12,13].

**Figure 1:**
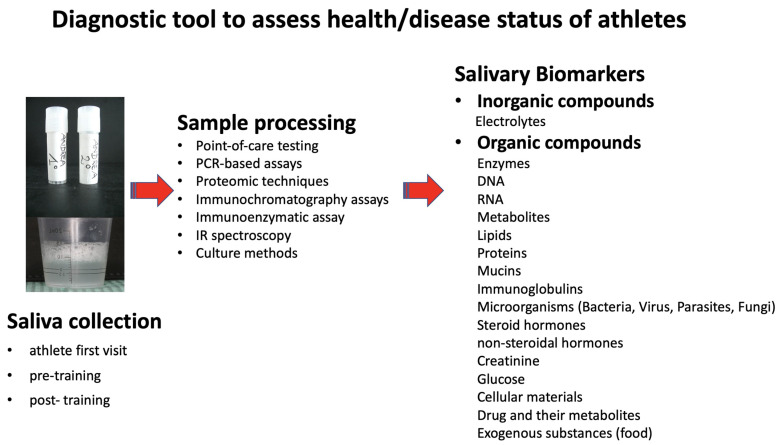
Human saliva collected from an athlete for the research of the main biomarkers.

Saliva hosts a broad spectrum of proteins/peptides, nucleic acids, electrolytes, heavy metals, microorganisms, hormones, drugs and neurotransmitters that come from multiple local and systemic sources. Indeed, human saliva can be defined as a “body mirror” because it can reflect the physiological and pathological conditions of the whole body, including the oral cavity [13,14,15]. Therefore, it can represent an important diagnostic and monitoring tool in many fields such as dentistry, allowing healthcare providers to assess athletes, health or disease status [16] (Figure 1).

Differences between individuals in the microbiological composition of saliva can also be reflected in variations in the biochemical composition. However, several authors agree that changes in the oral microbiota accompany a number of dental diseases, given the complex composition of saliva and its importance for oral homeostasis. Moreover, considering that the methods of collection and the degree of stimulation of salivary flow can influence the salivary composition, it is necessary to develop standard operating procedures for the collection of the sample itself and for the storage conditions [17].

Numerous valid reasons lead us to use saliva as a diagnostic fluid to monitor the state of health and disease. Human saliva offers several advantages over other biological fluids, such as serum, as it is readily available; quick and easy to collect, store and ship; low cost in sufficient quantities for the analysis; is not susceptible to transformations; and is easy to handle for diagnostic procedures. The sampling is cost-efficient and stress-free [15,16,18,19]. Saliva collection is less invasive and safer than venous sampling, which could expose patients and health care providers to infectious diseases such as HIV or hepatitis virus [20].

The use of saliva can be performed in the field of sports medicine and exercise to examine exercise-related endocrinological, immunological and microbiological status as well as to assess training loads and subsequently the risk of developing diseases [19].

For example, determining cortisol concentrations in sequentially collected saliva samples in response to circadian rhythms to assess exercise stress can diagnose and prevent overtraining syndrome (OTS) in athletes. Overtraining syndrome is an accumulation of training and/or non-training stress that causes a decrease in long-term performance capacity with or without signs and symptoms related to physiological and psychological maladjustment, in which the restoration of performance capacity requires several weeks or months [21]. Moreover, an evaluation of salivary immunoglobulins (S-IgA) and antimicrobial proteins (α-amylase, lysozyme, lactoferrin) allows one to determine the effects of exercise on mucosal immunity. Decreases in S-IgA and antimicrobial protein concentration and/or secretion rate have been described in athletes during a training season, thus making the athlete more susceptible to upper respiratory tract infections [19]. Sport activities allow athletes to have a more “health-associated” intestinal microbiota. It is characterized by a greater presence of bacterial species that promote health, greater microbial diversity, functional metabolic capacity and metabolites associated with microbes, which can modulate mucosal immunity and improve gastrointestinal barrier function [22].

Finamore et al. demonstrated a link between saliva and intestinal profiles in patients with inflammatory bowel diseases (IBD), and this result suggests that, even in athletes, saliva sampling can also be used as a biomarker for gut disease in the oral–gut axis [23].

By analyzing the saliva, in particular the pH values, buffer capacity, flow rate and growth of cariogenic bacteria, it is possible to assess caries risk [24,25,26]. Further possibilities for diagnosing specific oral and systemic diseases can also be obtained by saliva tests, such as the assessment of periodontal diseases and atherosclerosis by checking salivary inflammatory cytokines including IL-1β, IL-6, TNF-α and prostaglandin E2; acute myocardial infarction by evaluating the concentration of C reactive protein (CRP); and pancreatic cancer by searching for the presence of *Neisseria elongate* and *Streptococcus mitis* [25,26,27].

At the same time, as a diagnostic tool, saliva has some disadvantages. Among the limits to the wide use of saliva, the great variability of its normal composition, attributable to diurnal/circadian variations of some biomolecules contained therein, must be regarded. Based on these variations, particular attention should be paid to the sampling procedure and the fixing of the reference limits for the concentrations of the individual components. At the same time, the high variability in the composition of saliva can be exploited for the monitoring of various biorhythms (seasonal, close to 24 h, circadian, etc.) in order to study the physiological characteristics of the human body during sport activities [15,19,28].

Research based on the use of infrared spectroscopy technique, applied to detect circadian changes in the content of biological fluids, including saliva, has shown that gender- and age-dependent differences have been reported for some physicochemical characteristics of human saliva [29].

Currently, all doctors, dentists and laboratories should favor the use of saliva following the example of the American National Institute of Dental and Craniofacial Research (ANIDCR) that, in 2002, removed all obstacles and approved these body fluids as a diagnostic tool for assessing health and disease status [25,26].

**Figure 2:**
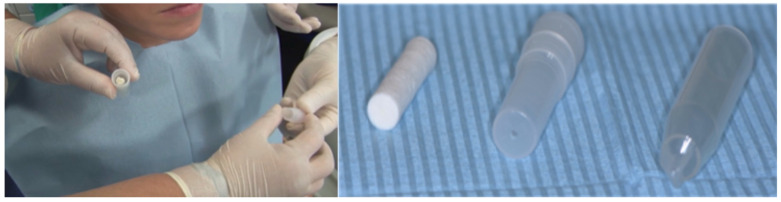
The collection of stimulated saliva with salivettes.

Thus, during the first visit of the athletes, salivary tests must be performed. The stimulated saliva can be collected with salivettes (Sarstedt AG & Co, Nümbrecht, Germany), with the athletes chewing the cotton roll present in the salivette for 1 min (Figure 2). The collected saliva can be subjected to chairside or laboratory analysis, in order to obtain information on microbiological, immunological and other ecological factors [30].

Moreover, the dentist must motivate the patient and can improve his compliance by determining the status of oral health through the saliva analysis. The checks must be continued during training, and must include new salivary tests for control of the variations in the oral ecological factors caused by sports activities and by use of the mouthguard.

Such attention to the starting situation and the changes that occur in the athletes’ oral cavity is justified by the need to resort to all available forms of prevention in order to preserve the oral health of athletes, usually adolescents.

## 3. Sports and Oral Health

The environment of the oral cavity is influenced by lifestyle, hygiene and eating habits, the possible intake of drugs and the performance of sports activities. Thanks to numerous researches carried out by sports dentists over the years, knowledge about the factors that regulate the oral ecology, such as salivary pH and flow, microbial load and S-IgA levels has been enlarged.

Firstly, diet, weekly frequency and training hours, climatic conditions and psychophysical stress conditions can produce significant variations in the oral ecosystem [1].

A review of the correlation between physical activity and oral health shows that the second seems to be low in a wide range of sports, and it is interesting to note how this condition is common in all competitive athletes. The sport can be considered one of the causes for bad oral health appearing in athletes [31,32].

The main diseases described frequently in the athletes’ oral cavity are caries, erosions and periodontal disease and several authors have analyzed the oral changes correlated to sports training. Needleman et al. (2015), examining young professional athletes, found that sport activities can be considered a risk factor among athletes from different sports for the onset of oral diseases, such as caries with an incidence between 15% and 70%; dental trauma 14–70%, dental erosion 36%, pericoronitis 5–39% and periodontal disease up to 15% [31,33,34,35].

Poor oral hygiene and all the physiological changes that occur during sport activities are fundamental factors in the development of oral and systemic diseases. The role played by oral bacteria is well known in cardiovascular diseases (atheromatous lesions, coronary disease), in esophageal cancer, colorectal and pancreatic cancer, as well as in rheumatoid arthritis [36].

Between several sport activities, football and swimming are the sports categories more at risk.

Gay Escoda et al., in a study on FC Barcelona players, reported values of the decayed, missing and filled teeth index (DMFT) that were used to evaluate caries prevalence of 5.7 and plaque index (PI), to evaluate oral hygiene according to Quigley and Hein, of 2.3 [37].

D’Ercole et al. demonstrated that young footballers had a salivary microbial load, relative to cariogenic microorganisms (such as *S. mutans*, *Lactobacillus* spp.), statistically higher than in boys who did not practice any sport, both before and at the end of sports training. A statistically significant decrease in S-IgA concentration also occurred after the training time [38].

These microbiological and immunological alterations expose young footballers to an increased risk in developing oral disease than sedentary subjects. In fact, the football players demonstrated a higher PI, an increase in dental discolorations, a higher frequency of bad habits (atypical swallowing, onychophagy) and a lower frequency of daily brushing in contrast to individuals who do not practice any kind of sports activity [38].

The training time is characterized by greater salivary function and intense physiological response. The decrease in the production of S-IgA and the association with an important intraoral growth of pathogenic bacteria leads us to consider the training period an open window for exposure to oral cavity diseases [39].

During exercise, intense physical activity produces lactic acid, which leads to a lowering of blood pH. A decrease in pH was also reported in the salivary level, before and after training. Changes in salivary pH are related to the level of CO2 in the blood: the levels of CO_2_ in the blood increase with the sport performance and, consequently, a high concentration of CO_2_ is transferred from the blood to saliva, with a consequent decrease in salivary pH [40].

Regarding the swimmers, the most common diseases reported are dental stains, caused by disinfectants of the pool water and by the time spent within the swimming pools. Although more than 6 h of training are required to develop them, they represent an esthetic problem with psychological repercussions; therefore, their onset must be considered as a health problem [32]. In addition, swimmers and water polo players are exposed to the onset of erosive tooth wear (ETW), a chemical-mechanical process characterized by a cumulative loss of hard dental tissue of a non-bacterial nature. This is a painful, irreversible condition and it is linked to the low pH values of the pool water (range 2.8–4.5), in contrast to normal values, equal to 7.2–8.0, or to an inadequate chlorination of the pool water [41,42].

Dental erosion is a common pathology among athletes and it can be correlated to the high consumption of food and drink in young people and sportspeople.

The widespread use, during sports activities, of sugary drinks with a very low pH, often lower than 3, depends on the spread of the internet and social media, that promotes the use of energy drinks, based on electrolytes and carbohydrates, in order to compensate hypoglycemia, dehydration, depletion of mineral salts and muscle glycogen that occur in athletes during physical activity. Indeed, the use of nutritional supplements is not supported by valid scientific literature and a healthy diet does not need mineral supplements [43,44,45].

The multifactorial nature of the pathogenesis of erosion in regards to both the use/abuse of energy drinks, takes into account the variables between the different drinks, the frequency with which they are introduced, the buffering power of saliva at the time of intake, the type of diet, the level of hydration and the degree of oral hygiene of the athletes [40,46,47,48,49,50].

Performing sports such as swimming in a well-controlled way and with adequate training can, on the contrary, induce benefits to the athlete.

D’Ercole et al., in competitive swimmers, showed a statistically significant decrease in S-IgA values, but their mean values were significantly higher than in non-competitive swimmers. Moreover, the cariogenic bacteria were present in the saliva of competitive swimmers with lower frequency than the “protective” microorganisms such as *S. sanguinis*, thus exposing athletes to a lesser risk of attracting caries, as confirmed clinically by lower values of active caries [2].

## 4. Sport Mouthguard: Effects on the Oral Ecosystem

Competitive athletes follow challenging training regimes to obtain optimum performance. Although sports activities have beneficial effects to general health, they also expose the athlete to an onset of oral diseases, including dental trauma; regardless of their origin (school, work, play, violence or accidental), they reflect on patient’s quality of life and require immediate treatment and a program of prevention of any future consequences.

The mouthguard is an oral device that, when inserted in the mouth, covers the palate and all occlusal surfaces of the teeth, to reduce oral–maxillofacial trauma and to protect the oral hard and soft tissues from fractures and lacerations and the maxillaries from fractures and dislocations [51,52].

The American Standards for Testing Materials classifies mouthguards into three categories: stock, boil and bite and custom-made [52].

Ethylene vinyl acetate (EVA) is the most suitable material for the mouthguard. It is a polymeric plastic of ethylene and vinyl acetate, characterized by flexibility, elasticity and certified biocompatibility [52,53].

The primary function of the mouthguard is trauma prevention; however, this device can be utilized for other functions, for example, as a reservoir of materials designed to prevent oral diseases, such as chlorhexidine, fluorine, casein. Moreover, it can be a valid aid in the athlete’s performance, as it provides a psychological and a physical advantage.

Chiavaroli et al. showed an inhibition of 8-iso-prostaglandin_2α_, a marker of antioxidant deficiency and lipid peroxidation, in salivary release in athletes who used the mouthguard during the game. In contrast, the authors described a significant increase in the salivary isoprostane level in athletes who did not use the mouthguard [54].

Recent studies showed that the use of the mouthguard improved physical performance by increasing, in both aerobic and anaerobic activities, the respiratory capacity of athletes [55]. In subjects with reduced respiratory capacity, as in patients with cystic fibrosis, 8-iso-PGF_2α_ concentration was significantly higher than in healthy controls [56]. Based on results obtained, Chiavaroli et al. hypothesized that the use of the mouthguard increases aerobic performance and leads to a further decrease in 8-iso-PGF_2α_ release with a reduction in oxidative stress [54].

Considering numerous advantages, custom-made mouthguards, conceived and realized by specialized dentists and dental technicians to guarantee the characteristics of protection, stability, individuality and comfort, must be adopted in all sports activities.

Despite the obvious benefits of using the mouthguard, including the possibility of overcoming communication, hydration and nutrition difficulties, the clinician should not overlook other problems that may arise from its use, especially in professional sportsmen who undergo several hours of training every day.

In fact, inadequately designed, worn and jagged-edged mouthguards can result in injuries to the oral mucosa, such as hyperkeratosis, erythema and ulceration, as described in the mouths of athletes who had worn boil and bite mouthguards for an entire competitive season. Several authors reported that the wounds caused by damaged mouthguards, at the oral level, can favor the entry of microorganisms present on the surface of the mouthguard into the bloodstream, with consequent opportunistic systemic infections (eg., endocarditis, pericarditis, pneumonia, etc.) [57,58].

As described by Glass et al., the boil and bite mouthguards can be contaminated by several microorganisms and therefore can become a microbial reservoir from which can be generated oral and systemic diseases [59].

“Boil and bite” mouthguards, worn by hockey and football players, harbored many pathogenic and opportunistic bacteria, yeasts and molds. The main bacterial species found on mouthguard surfaces were: *Staphylococcus* spp., *Micrococcus* spp., *Brevibacterium* spp. and *Cellulomonas* spp. The most common species of yeasts were *Candida parapsilosis* and *Rhodotorula mucilaginosa*, while the most common species of mold were *Bipolaris*/*Cochliobolus* spp. and *Penicillium chrysogenum* [58,60].

Batoni et al. reported that the use of oral removable devices creates new retention niches that favor *S. mutans* biofilm colonization [61].

Glass et al., with the aid of SEM, hypothesize that the pathogenic change of the microorganisms on the surface of the mouthguard may depend on the material wear and tear and on the difficulty in preserving it [58,59].

D’Ercole et al., in a study performed to monitor changes in the oral cavity by determining clinical, salivary and bacterial markers, before, during and after sports treatment with custom-made mouthguard, confirm that the use of these dental devices is among the factors that can affect oral homeostasis [53].

They reported that the prolonged use of the device elevates full mouth plaque score (FMPS) and full mouth blood score (FMBS) and decreases the buffering capacity and salivary pH, thus inhibiting the saliva protective effect [53]. In this study, Saliva-Check Mutans GC^®^ test demonstrated that the microbial load of *Streptococcus mutans* remained unvaried during the observation period, according to Sanpei et al. and Lara-Carrillo et al. In addition, one year of therapy leads to a worsening of the patient’s clinical indexes, because they lose the motivation to maintain good oral hygiene [62,63].

## 5. Sport Mouthguard in the Prevention of Oral Diseases

Competitive players can achieve high performance only in the presence of a state of health.

Boil and bite mouthguards are the most used by athletes for their low cost and easy access but at the same time they have numerous disadvantages.

Instead, it is important to emphasize that custom-made mouthguards have many advantages over stock and ready-made: they show optimum comfort and a good fit and they reveal no negative effects on faction satisfaction of elite Taekwondo athletes. Moreover, they offer protection against orofacial injuries and allow stable muscular activity during the training of Karate-Dō athletes [52,64].

**Figure 3:**
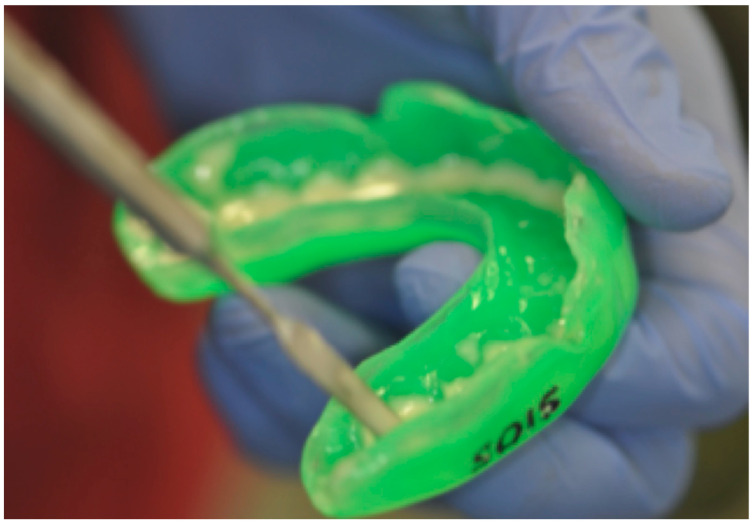
Addition of protective substances inside the device to prevent oral damage caused by the mouthguard.

To prevent oral damage caused by the prolonged use of mouthguard and the adhering pathogenic microorganisms, some strategies can be used, such as addition of protective substances inside the device (Figure 3). The chlorhexidine can be used as an active ingredient for preventing and reducing the colonization, development and pathogenicity of dental plaque [65].

The use of chlorhexidine in the mouthguard was encouraged by the effects obtained from D’Ercole et al. in an in vivo study. From this study emerged the observation that the addition of chlorhexidine, during the training time, inhibits microbial proliferation on the support itself and reduces the salivary concentration of *Streptococcus mutans*, *Candida* spp. and molds. Moreover, the contemporary use increases the value of the salivary pH and buffering capacity [66,67].

According to the different needs of the patient, other materials such as fluoride and casein can be used.

Fluoride is indicated for patients who need a fluoroprophylaxis protocol, which involves the use of fluoride (toothpaste and/or gel) to strengthen dental enamel and to make it less susceptible to bacterial attack and to the formation of caries [1].

Research on casein showed that its application in custom-made EVA mouthguards is able to directly counteract the adhesion of plaque on the surface of the teeth and to increase pH values, salivary flow, amount of stimulated saliva and buffer capacity, improving the oral health of the athletes [68].

The positive effects on the white spot lesions, the increase of salivary flow, buffering capacity and pH values, are referred to by Hegde and Thakkar, through the use of casein-chewing gums [69].

Nagai et al. propose to insert a bioactive filler into the structure of the EVA material. This new material proved to be bacteriostatic against *Streptococcus mutans* and *Porphyromonas gingivalis* and it has no cytotoxic effect on human gingival cells [70]. Yoshida et al. showed that silver nanoparticles, embedded in the EVA matrix, exhibited effective antibacterial properties against *Streptococcus sobrinus, Porphyromonas gingivalis* and *Escherichia coli*, thus suggesting the use of this material for the construction of mouthguards [71].

Sadly, bacteria, saliva and food debris tend to accumulate on the mouthguard, giving it an unpleasant smell and taste, thereby requiring constant cleaning of the device [59].

Following the basic rules of oral hygiene before exercise and cleaning the device after each use can prevent damage to the oral cavity. Unfortunately, athletes tend to easily neglect the rules of conservation and cleaning of the mouthguard. Namba et al., in an interview given by 22 rugby players, showed that only two of them were properly guided by a sports dentist on ways to sanitize their mouthguard [72].

There are still no precise and well-coded rules for the maintenance of mouthguards and few authors have described them. Barton recommended daily disinfection by immersion in a commercially available antimicrobial denture-cleansing solution [73]. Ogawa achieved efficient hygienic storage of EVA dental devices by washing them with sterilized water and storing them in a ventilated environment [74].

D’Ercole et al., testing different disinfectants, demonstrated that there is no ideal method. The solutions such as hydrogen peroxide, 0.5% sodium hypochlorite and Oral Care Foam™ resulted in a significant reduction of microorganisms adhering to the surface and achieved optimal mouthguard disinfection. SEM observation confirmed that the different substances, indicated above, reduce the development of microbial communities on the EVA surface [67].

In general, the athlete must still be advised to carry out daily hygiene of the mouthguard with appropriate antimicrobial solutions; not to leave the device soaking in the detergent solution for a long time, to prevent it from taking on a bitter taste and acquiring a viscous surface film; not to immerse it in mouthwashes or solutions containing alcohol, to avoid deterioration of the material; and, finally, to rinse the mouthguard dipped in the cleaning solution before inserting it into the mouth [66,73,74].

As for storage, many athletes do not care about the possible contamination of the mouthguard and deposit it everywhere. The most suitable method for storage remains the use of perforated containers that allow ventilation, after drying the mouthguard with a napkin to remove liquid residues.

Moreover, it is necessary to specify that mouthguards are made with a highly deformable plastic material when exposed to high temperatures [75].

For this reason, it is recommended not to dry it with a hairdryer, not to use boiling water and to avoid exposure to direct sunlight for long periods of time.

## 6. Conclusions

Studies carried out on effects of sports activities on the oral cavity are different and concern several aspects of this issue. Although sport is an absolutely healthy and positive habit in the life of the young or adult people, it can be considered a lifestyle at risk for development of various pathologies.

These risks can be avoided by using a custom-made mouthguard due to its triple function: protection against sports-related injuries; reservoir of substances protective for the oral ecology; improvement of athletic performance.

Moreover, monitoring the athletes with oral health screening, clinical examination, salivary analysis and implementing oral health promotion programs may provide an assessment of sport athletes’ risk status for developing several diseases.

The study of microbial markers, immune status and habits of sports subjects is essential to establish the management of the training load, with the aim of reducing physical stress, the risk of oral infection and a worsening of the quality of life (oral diseases affect self-esteem, nutrition and health, as they cause pain, anxiety and social discomfort).

Since the disease can stop exercise and performance, further research, to clarify how the sports training affects immunity and health status, is needed.

In the future, analysis of saliva could be applied in a personalized way to choose the best diet and training program for the individual to achieve the best possible performance and to prevent athletes’ injuries.

## Data Availability

No new data were created or analyzed in this study. Data sharing is not applicable to this article.
